# Impostor phenomenon and its association with perceived stress and anxiety among students in medical and social sciences at a Swedish university

**DOI:** 10.3389/fmed.2025.1623792

**Published:** 2025-10-29

**Authors:** Anna Jansson, Jens Boman, Ingrid Schéle, Stefan Holmström, Alexander Rozental, Olof Semb, Martin Fahlström, Laura Stenman, Aziz Bitar, David Lindquist

**Affiliations:** ^1^Department of Clinical Sciences, Professional Development, Umeå University, Umeå, Sweden; ^2^Department of Psychology, Umeå University, Umeå, Sweden; ^3^Department of Health, Education and Technology, University of Technology, Luleå, Sweden; ^4^Department of Clinical Neuroscience, Karolinska Institutet, Stockholm, Sweden

**Keywords:** dental, law, medical, nursing, psychology, university students, impostor phenomenon, imposter syndrome

## Abstract

**Introduction:**

Psychological distress is of concern among university students worldwide, more so than in a comparable working population. The impostor phenomenon (IP) describes feelings of inadequacy often experienced by individuals struggling to internalize success despite evidence to the contrary. IP is prevalent among university students and has been identified as a significant factor in understanding psychological distress within this population. This study aimed to investigate the prevalence of IP and its association with perceived stress and anxiety in dental, law, medical, nursing and psychology university students.

**Methods:**

A web-survey consisting of the Clance Impostor Phenomenon Scale (CIPS), the Perceived Stress Scale-4 (PSS-4), the Generalized Anxiety Disorder-2 (GAD-2), and sociodemographic questions were completed by 968 university students registered at a Swedish university during 2022 and 2023. The prevalence of IP, perceived stress, and anxiety was calculated. Pearson’s correlation coefficient and multiple linear regression were used to examine the relationship between the variables.

**Results:**

64.0% of the participants scored above the cut off value for experiencing IP (CIPS score ≥62). According to cut-off levels developed to categorize the intensity of IP experiences 8.4% of participants had low experiences of IP, 26.0% moderate, 42.6% frequent, and 23.0% intense experiences of IP. Of all participants, 91.6% had at least moderate experiences of IP and 65.6% had frequent to intense experiences of IP. Women scored significantly higher on CIPS than men. In contrast, neither attending semester nor age group significantly impacted CIPS scores. Finally, there was a moderate correlation between the levels of perceived stress and anxiety, respectively, and the IP scores.

**Conclusion:**

This study suggests that the majority of dental, medical, nursing, psychology and law students experience severe IP. Moreover, this study provides valuable insights into the association of IP with perceived stress and anxiety. The results underscore the significance of exploring IP and its link to psychological distress, suggesting that interventions aimed at diminishing IP may play a crucial role in enhancing the well-being of university students.

## Introduction

Studying at the university is often socially and academically demanding, and consequently, many students can feel pressured. Over time, high levels of stress can lead to psychological distress, including anxiety and depressive symptoms. Psychological distress is a concern among university students worldwide, more so than in comparable working populations (1–4), which is true also for university students in Sweden (5, 6). The impostor phenomenon (IP) (7) has been shown to be relevant for explaining psychological distress among university students, but the prevalence remains unclear.

IP is a psychological pattern recognized and described in 1978 by Clance and Imes in their study of high-achieving women, who believed they were not deserving of their success (8). IP is not a clinical diagnosis but rather a psychological experience that affects how individuals perceive their skills and successes. Individuals with IP experience deliberating self-doubt, often leading high-achieving individuals to ascribe their success to external factors such as luck or the errors of others instead of their own abilities (9). IP could motivate and increase their drive to succeed for some individuals, positively impacting their careers (10). However, most studies on IP have focused on the negative impact IP has on academic success and mental health, as individuals who experience IP struggle with uncertainty and feelings of inadequacy. They may have an irrational fear of being exposed as a fraud, which can hinder their ability to recognize their potential and achieve their goals. This can be a significant obstacle that prevents individuals from fulfilling their potential (11). Individuals with IP also experience higher levels of negative feelings and dissatisfaction with their work (12). IP has been linked with high levels of emotional exhaustion at work and increased work–family conflict (13). IP is also associated with perfectionism and high levels of work-related stress (14) and has also been linked to burnout (11, 15). Furthermore, IP has been associated with psychological distress, including but not limited to perceived stress (16, 17), anxiety (17–21), depression (17, 19–21), and increased suicidal ideation and suicide risk (16, 22). Even though Clance and Imes initial research focused on women, later studies have shown that it affects both men and women (7).

The reported prevalence of IP varies between 9 and 89% in the literature (7). Approximately half of the studies investigate IP among students, the other half among professionals (7). Studies concerning student populations, most conducted in North America and in the field of health care, have shown a high prevalence of IP among various university student programs (20, 23–27). To our knowledge, only a few studies investigate IP among students from the faculty of social sciences (28, 29), and so far only one study has investigated IP among students in a Swedish context (30). Furthermore, international studies show that IP has negative consequences for the well-being of university students that can be sustained even in their lives as professionals (7). Therefore, it is also necessary to investigate IP and its relation to psychological distress among university students in a Swedish context.

This study aimed to investigate the prevalence of IP in dental, law, medical, nursing, and psychology students at a large university in Sweden. These programs were in part selected because of their high admission scores, and they represent both the faculties of medicine and social sciences. Furthermore, they are all vocational education programs. A second aim was to investigate the association between IP and perceived stress and anxiety.

## Methods

### Study design

The present study was part of a larger project exploring students’ well-being at Umeå University, Sweden. The study design was cross-sectional, aiming to investigate the prevalence of IP and its relation to perceived stress and anxiety.

### Measures

An online survey was created using Microsoft Forms, containing demographic questions, such as age group, gender, and current semester, and scales measuring IP, perceived stress, and anxiety.

Clance Impostor Phenomenon Scale (CIPS) is a 20-item validated instrument measuring an assortment of traits associated with IP, such as the fear of failure, attribution of success to luck, and fear of not being able to repeat success (31, 32). A Swedish version of CIPS, that was translated into Swedish and then back translated into English was used in this project (30). The validation of the Swedish version is in progress. Approval to use CIPS was obtained from Dr. Pauline Rose Clance. Each item is scored on a five-point Likert Scale, receives a point score from 1 to 5, and the sum of responses to the individual items is used to create an aggregate score. The total score ranges from 20 to 100, with higher scores indicating greater IP experience. Cut-off levels have been developed to categorize individuals based on aggregate scores into few (40 or less), moderate (41–60), frequent (61–80), and intense (81–100) IP experiences (31). The scoring system of the CIPS questionnaire has been further defined by Holmes and colleagues (32), where a total score of 62 was used as a cut-off value to distinguish impostors (≥62 = high level) from non-impostors (<62 = low level).

The four-item Perceived Stress Scale-4 (PSS-4) is a short version of the Perceived Stress Scale (PSS) that evaluates (positive and negative elements in) the perception of (and coping with) stress (33). Each item receives a point score from 0 to 4, and the sum of the responses to the individual items is used for the aggregated score. The total score ranges from 0–16. There are no established cut-offs; however, a score between 0–8 indicates normal levels of perceived stress, and 9–16 indicates elevated levels. The English and the Swedish versions of PSS-4 have been validated (34, 35).

The two-item Generalized Anxiety Disorder-2 (GAD-2) is a short form of the Generalized Anxiety Disorder Scale-7 (GAD-7), which aims to find those at risk of anxiety disorders (36). The two items in GAD-2 receive scores from 0 to 3, and the sum of the responses to these items is used to create an aggregated score. The total score ranges from 0–6, where a score of 3 or more can identify clinically significant anxiety levels. The GAD-2 scale has been validated (37) and has been shown to retain the psychometric properties of GAD-7 (*α* = 0.92 and intraclass correlation = 0.83) (36).

### Data collection and participants

Students from five different programs at Umeå University in the northern part of Sweden were invited to participate in the study. Data was collected in two waves. In the first wave, during the spring semester of 2022, data from students in dental, law, medical, and nursing programs were collected. In the second wave, during the autumn semester of 2023, data from students in the clinical psychology program were collected.

Information about the study and the survey link were circulated through each program’s mailing lists provided by study administrators and shared through private social media groups connected to the university. According to the Helsinki Declaration, all students were informed that participation was voluntary and that their anonymity and confidentiality were assured.

Of the 2,430 invited students, 968 responded, resulting in a response rate of 37%. Half of the study population (*n* = 488, 50%) were medical students, and the students from the dental, law, nursing, and clinical psychological programs were distributed among the other half of the study population. Among the invited students, 70% were women; in the study population, women comprised the majority of the sample (*n* = 682, 71%). The gender distributions in percentage (W/M) among the students in the different programs were 59/41 in the medical program, 66/34 in the dental program, 86/14 in the nursing program, 72/28 in the clinical psychology program, and 63/37% in the law program. The gender distribution in percentage (W/M) among the study participants in the different programs were 65/35 in the medical program, 73% women in the dental program, 88% women in the nursing program, 74% women in the clinical psychology program, and 70% women in the law program.

Most of the students in the study population were 29 years or younger (*n* = 838, 86.6%).

The participants represented the beginning, middle, and end of each program. The medical program’s students represented all eleven semesters (data not shown). The demographics of the participants are presented in Table 1.

**Table 1:** A descriptive table of the population, divided by low, high, and four different levels of Impostor phenomenon.

Variables	Non-impostors (20–61) *N* (%)	Impostors (62–100) *N* (%)	Few (20–40) *N* (%)	Moderate (41–60) *N* (%)	Frequent (61–80) *N* (%)	Intense (81–100) *N* (%)	Total (100%)
Total	348 (36.0)	620 (64.0)	81 (8.4)	252 (26.0)	412 (42.6)	223 (23.0)	968
Program							
Medical	189 (38.7)	299 (61.3)	53 (10.9)	127 (26)	197 (40.4)	111 (22.7)	488
Nurse	28 (19.2)	118 (80.8)	5 (3.4)	21 (14.4)	78 (53.4)	42 (28.8)	146
Dental	43 (44.8)	53 (55.2)	5 (5.2)	38 (39.6)	35 (36.5)	18 (18.8)	96
Psychology	62 (44.3)	78 (55.7)	14 (10.0)	45 (32.1)	60 (42.9)	21 (15.0)	140
Law	26 (26.5)	72 (73.5)	4 (4.1)	22 (22.4)	41 (41.8)	31 (31.6)	98
Gender							
Female	211 (30.9)	471 (69.1)	42 (6.3)	159 (23.3)	302 (44.3)	178 (26.1)	682
Male	130 (47.1)	146 (52.9)	38 (13.8)	86 (31.2)	108 (39.1)	44 (15.9)	276
Nonbinary	7 (70)	3 (30)	0	7 (70)	2 (20)	1 (10)	10
Age							
<30	302 (36)	536 (64)	63 (7.5)	225 (26.8)	357 (42.6)	193 (23.0)	838
≥30	46 (35.4)	84 (64.6)	18 (13.8)	27 (20.8)	55 (42.3)	30 (23.1)	130
Perceived stress							
Normal	314 (43.7)	405 (56.3)	77 (10.7)	225 (31.1)	291 (40.5)	126 (17.5)	719
Elevated	34 (13.7)	215 (85.3)	4 (1.6)	27 (10.8)	121 (48.6)	97 (39.0)	249
Anxiety							
Normal	264 (45.2)	320 (54.8)	74 (12.7)	181 (31)	241 (41.3)	88 (15.1)	584
Elevated	84 (21.9)	300 (78.1)	7 (1.8)	71 (18.5)	171 (44.5)	135 (35.2)	384

Impostor phenomenon measured by Clance Impostor Phenomenon Scale. Perceived stressed measured by Perceived Stress Scale-4. Anxiety measured by Generalized Anxiety Disorder-2.

### Statistical procedure

From Microsoft Forms, data was transferred to Microsoft Excel, where items were sorted, recoded and labelled before being imported to SPSS (Version 28.0, IBM Corp). The material had <0.1‰ missing values, and we used the mean value for the actual observed data to fill in for missing values. Descriptive statistics were used to characterize the sample. The group of participants who defined themselves as non-binary gender was too small to include in the statistical analysis. Prevalence of self-reported IP, perceived stress, and anxiety in all student groups were primary outcome measures. For secondary outcome independent *t*-tests were used for calculating differences in mean data between age groups and gender and the scales used in the study. Pearson’s correlation coefficient was used to examine the association between CIPS scores and PSS-4 and GAD-2. Multiple linear regression models were constructed with PSS-4 and GAD-2 scores as the dependent variables, respectively, and gender, age group, and CIPS score as independent variables. The level of significance was set at *p* < 0.05.

### Ethical considerations

The participants provided their informed consent to participate in the study by returning their completed questionnaires. The questionnaire was anonymous and voluntary, and no personal information was collected. Therefore, the study was considered exempt from requirements for approval by the Swedish Ethical Review Authority under The Swedish Act (2003:460) concerning the ethical review of research involving humans.

## Results

Table 1 provides sample demographic characteristics divided by the level of IP.

### Prevalence of impostor phenomenon, perceived stress, and anxiety

**Figure 1:**
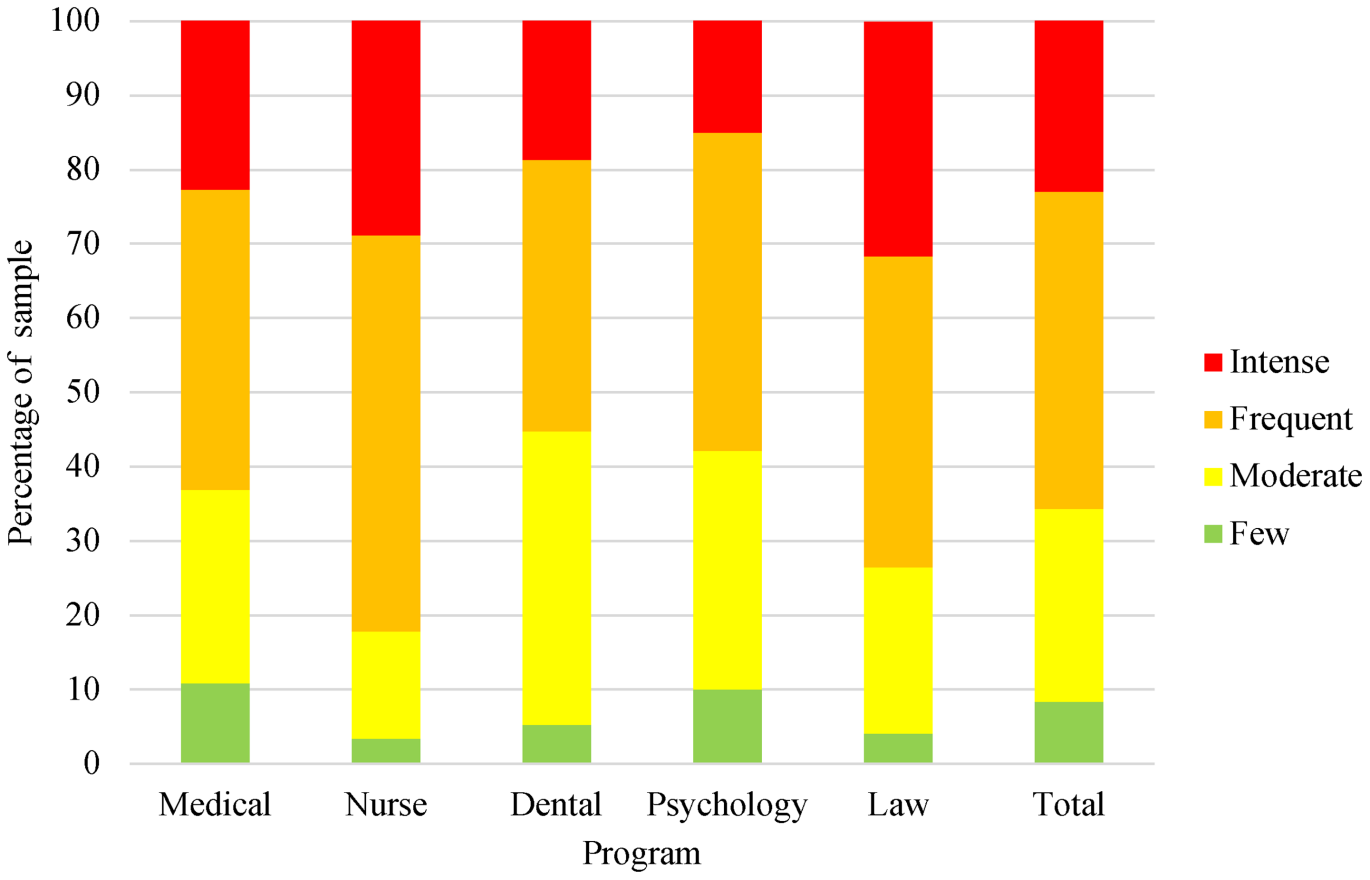
Distribution of Clance Impostor Phenomenon Scale scores across academic programs. IP measured by Clance Impostor Phenomenon Scale, divided in four different levels.

Of all the participants, 620 (64.0%) scored above the CIPS cut-off value for experiencing IP and 635 (65.6%) scored frequent or intense impostor feelings, as shown in Figure 1. The overall mean scores for CIPS were 66.73 (SD ± 16.61), as shown in Table 1.

Table 1 shows that a greater number of students exhibited heightened levels of anxiety and perceived stress within the group of participants scoring above the cut-off value for experiencing IP compared to the participants scoring under the cut-off value.

Independent *t*-tests were conducted to compare mean scores of PSS-4 and GAD-2 between participants scoring above and below the IP cut-off, as shown in Table 2. The results show a significantly higher level of perceived stress and anxiety among those scoring above the IP cut-off.

**Table 2:** Perceived stress and anxiety in participants with low (non-Impostor) and high levels of Impostor phenomenon (Impostor).

Variables	Non impostor		Impostor		*t* (966)	*p*	Cohen’s *d*
	*M*	*SD*	*M*	*SD*			
Perceived stress	5.08	2.67	7.23	2.70	−11.927	<0.001	0.80
Anxiety	1.75	1.68	2.91	1.82	−9.79	<0.001	0.66

Impostor phenomenon measured by Clance Impostor Phenomenon Scale. Perceived stressed measured by Perceived Stress Scale-4. Anxiety measured by Generalized Anxiety Disorder-2. Mean parameter values for each of the analyses are shown for low IP scores 21–61 (*n* = 348) and high IP scores 62–100 (*n* = 620), as well as the results of *t* tests (assuming equal variance) comparing the parameter estimates between the two levels of IP.

To investigate gender differences, an independent *t*-test was conducted to compare mean scores of CIPS, PSS-4 and GAD-2 scores, as shown in Table 3. The results show a significantly higher level of IP, perceived stress and anxiety in women compared to men.

**Table 3:** Gender difference in the mean score of the included variables in the questionnaire.

Variables	Women		Male		*t* (956)	*p*	Cohen’s *d*
	*M*	*SD*	*M*	*SD*			
Impostor phenomenon (IP)	68.69	16.22	62.12	16.76	5.627	<0.001	0.40
Perceived stress	6.70	2.82	5.87	2.96	4.023	<0.001	0.29
Anxiety	2.69	1.85	2.00	1.78	5.295	<0.001	0.38

Impostor phenomenon measured by Clance Impostor Phenomenon Scale. Perceived stressed measured by Perceived Stress Scale-4. Anxiety measured by Generalized Anxiety Disorder-2. Mean parameter values for each of the analyses are shown for women (*n* = 682) and men (*n* = 276), as well as the results of *t* tests (assuming equal variance) comparing the parameter estimates between the genders.

Independent *t*-tests were also conducted to compare CIPS scores between women and men in the different university programs. In the medical, dental, and clinical psychology programs, women showed a significantly higher CIPS score than men. However, in the nursing and law programs, the mean CIPS score was higher in men, although the difference was not significant.

The independent *t*-test did not show a significant difference in the CIPS scores between age groups.

A one-way ANOVA was conducted to compare the mean CIPS scores between the different programs. The results revealed significant differences in CIPS scores between the programs, *F*(4, 963) = 9.435 *p* < 0.001 with a small effect size (*η*^2^ = 0.038). Tukey’s HSD *post hoc* test showed that the students from both the nursing and law programs scored significantly higher than the students from the medical, dental, and clinical psychology programs (*p* < 0.001).

A one-way ANOVA was conducted to compare the mean CIPS scores between the different semesters in the medical program but did not show any significant differences. Since study participants were not represented in all semesters in the other programs, this calculation was only made for the medical program.

### Relationship between variables

A Pearson’s Correlation coefficient was used to examine the relationships between IP, perceived stress, and anxiety among all study participants. IP was positively correlated to stress, *r* (968) = +0.444 *p* < 0.001 and to anxiety, *r*(968) = +0.383 *p* < 0.001. A complete list of correlations is presented in Table 4.

**Table 4:** Means, standard deviation, and Pearson correlations matrix for continuous variables (*n* = 968).

Variables	M	SD	1	2	3
1. Impostor phenomenon (IP)	66.73	16.61	−	−	−
2. Perceived stress	6.46	2.88	0.444**	−	−
3. Anxiety	2.49	1.85	0.383**	0.666**	−

**p < 0.001. Impostor phenomenon measured by Clance Impostor Phenomenon Scale, Cronbach’s alpha = 0.92. Perceived stress measured by Perceived Stress Scale-4, Cronbach alpha 0.74. Anxiety measured by Generalized Anxiety Disorder-2, Cronbach alpha 0.86.

Multiple regression analyses were conducted to evaluate the extent to which gender, age and IP could predict perceived stress and anxiety, respectively.

With perceived stress as the outcome variable a significant regression was found (*F*3,964 = 79.72, *p* ≤ 0.001). The *R^2^ adjusted* was 0.196, indicating that IP explained approximately 19.6% of the variance in perceived stress (Table 5).

**Table 5:** Effect of gender, age, and Impostor phenomenon on perceived stress.

Variables	Model 1 (gender, age)	Model 2 (gender, age, IP)
Gender, age	−0.055 (0.098)	−0.020 (0.089)
Impostor phenomenon (IP)	−	0.076 (0.05)
Intercept	6.763*** (0.187)	1.459*** (0.394)
Adjusted *R*^2^	0.013	0.196
Sample size (*N*)	968	968

***p < 0.001. Impostor phenomenon measured by Clance Impostor Phenomenon Scale. Perceived stress measured by Perceived Stress Scale-4. Unstandardized B-coefficients, coefficient standard-errors in brackets. Dependent variable perceived stress. Regression analysis.

With anxiety as the outcome variable a significant regression was found (*F*3,964 = 58.59, *p* ≤ 0.001). The *R^2^* adjusted was 0.152 indicating that IP explained approximately 15.2% of the variance in anxiety (Table 6).

**Table 6:** Effect of gender, age, and Impostor phenomenon on anxiety.

Variables	Model 1 (gender, age)	Model 2 (gender, age, IP)
Gender, age	−0.047 (0.063)	−0.007 (0.059)
Impostor phenomenon (IP)		0.041*** (0.003)
Intercept	2.747*** (0.120)	−0.126 (0.261)
Adjusted *R*^2^	0.022	0.152
Sample size (*N*)	968	968

***p < 0.001. Impostor phenomenon measured by Clance Impostor Phenomenon Scale. Anxiety measured by Generalized Anxiety Disorder-2. Unstandardized B-coefficients, coefficient standard-errors in brackets. Dependent variable anxiety. Regression analysis.

## Discussion

This study investigated the prevalence of IP and its association with perceived stress and anxiety among students from the dental, law, medical, nursing, and clinical psychology programs at a large university in Sweden. This study confirms that IP is highly prevalent among university students, as only 8.4% of the participants experienced few impostor feelings, while 65.6% experienced frequent or intense impostor feelings. Furthermore, a majority of the students (64%) scored above the cut-off value indicating IP. The regression analysis indicates that IP could explain the variance in perceived stress and anxiety with approximately 20 and 15%, respectively. Furthermore, this study suggests that IP is significantly associated with higher levels of perceived stress and anxiety.

### Considerable prevalence of IP among students in all the programs studied

The findings demonstrated a significant prevalence of IP, indicating that IP is common among Swedish university students. In line with previous research, the prevalence of IP in this study is higher than in the general population (38, 39). The high prevalence of IP shown in this study is similar previous studies on university students in the health area, as shown by Holiday et al. (40) and Rosenthal et al. (41). A review of IP in medical students reports a prevalence between 20 and 60% (7), and a review of IP in nursing and newly registered nurses report a prevalence between 36 and 75% (42). Only a few studies have been published on IP in law and psychology students, showing 34.6% prevalence in law students (26) and 56% prevalence in psychology students (43). In this study, more than 50% of the students in each of those programs scored above the cut-off level for IP. Surprisingly, students in the nursing and law programs showed higher prevalence in IP compared to the other programs. The law program differs in their four-grade system compared to the other programs having a three-grade system which might trigger impostor feelings even more (44, 45). However, the high prevalence in the nursing program is harder to explain. Possibly the effect of perceived minority status could be at play (46), as in this case the male students report more severe impostor feelings than their female peers, possibly related to the fact that traditional norms do not associate masculinity with nursing (47). We would however note that this is a minority by numbers.

The findings further demonstrated that the prevalence of IP remained stable across the entire medical program, which is consistent with some previous findings (30, 40, 41) on medical students, but in contrast to Villwock et al. (15) and Frenchi et al. (26) who found that the prevalence of IP was lower at the end of the medical program. Nonetheless, the cross-sectional design in this study prevents us from drawing further conclusions.

In addition, the results from this study did not show any significant differences in CIPS scores between the younger group (29 years old or below) and the older group (30 years old or above). Hence, it appears that all age groups report IP. This contrasts previous studies, that have shown that CIPS scores tend to decrease with increasing age (48). However, it is necessary to highlight that most study participants in this study belong to the younger demographic, complicating the analysis of age-related factors impacting IP.

### Gender differences in IP prevalence and psychological distress

In this material, the overall mean score of IP indicates that the average participant experienced frequent impostor feelings. When comparing the mean score of IP among all study participants, women exhibit a significantly higher prevalence of IP compared to men. The results align with some previously published international studies, where the results have been equivocal. While some found no gender differences in the IP prevalence among university students (9, 34), others reported women having a higher prevalence of IP compared to men (40, 49–51).

When examining gender differences in the different programs, the results show that the IP prevalence is significantly higher for women in the dentistry, medical, and clinical psychology programs but not in the law and nursing programs. Although not significant, the results indicate that IP may be higher for male students in law and nursing programs. The results from the nursing program are consistent with findings described by Henning et al. (3), where male nursing students reported higher levels of IP and more psychological distress compared to female nursing students. This could be explained by the fact that women historically dominate the nursing field, even today, with remaining stereotypes and expectations in the nursing profession. The gender difference could also be explained by the small sample size of male nursing students, causing a skewed result.

The female participants in this study scored significantly higher on perceived stress and anxiety when compared to their male counterparts. These results, combined with the higher average score of IP among the women, indicate a greater level of psychological distress in the female participants in comparison to the male participants, which is expected (5).

### Association between IP and psychological distress

In line with previously published international studies, the findings in this study indicate a relationship between IP and perceived stress and anxiety (16, 18–20). Individuals with elevated IP scores also score significantly higher levels of perceived stress and anxiety. The analysis on mean scores and the correlation underscores the relationship between IP and psychological distress in the study participants.

It was considered essential to assess the predictive ability of different parameters on perceived stress and anxiety. The regression analysis indicates, as expected, that IP significantly predicts perceived stress and anxiety. These results are consistent with previous investigations on students concerning IP and its association with psychological distress among students (22, 24, 52).

### Strengths and limitations

The current study is one of the largest studies reporting on IP among university students, involving students from the faculties of medical and social sciences. The results of this extensive study provide further insight into the prevalence of IP and its association with psychological distress among university students. Both the scales for measuring anxiety and perceived stress are validated, though the Swedish version of CIPS is under validation. Though good internal consistency (Cronbach’s alpha 0.92) the ongoing validation study conducted on this sample has not successfully replicated the commonly reported one-factor solution (just 25% explained variance). This should not be a problem as the theoretical foundations of IP stipulate three factors – fake, disregard and luck. However, we find that several previous validation studies published incomplete information regarding what items constitute what construct, an issue previously raised (53), which conclusion that rather than relying on factor analysis, an approach that focuses on how that items are understood and perceived in a Swedish context, e.g., a Delphi study (54) is the best way forward. Even though the participants represent students from medical and social sciences faculties and are distributed across the entire curriculum, the respondents may not adequately represent the overall student body in the medical and social sciences in Sweden. The study’s cross-sectional design, based on self-reported data, possesses several strengths, but some limitations need to be considered. In a cross-sectional design effects must be interpreted with caution, though the theory of the IP states that it is established during childhood (8), thus implying a chronology. As there are possible overlapping constructs, such as fear of failure (55), the relation between CIPS and adjacent scales needs to be examined more thoroughly. Although web surveys appear convenient, response rates tend to be on the lower side and some recommendations – such as keeping the survey short (56) are hard to adhere to while also validating an instrument. Our choice to conduct a completely anonymous survey ruled out targeted reminders. Moreover, survey response rates appear to be on the decline in society in general, one possible explanation being an increase in survey requests (57) though this trend might not necessarily skew the data (58). Also, lower health and wellbeing appear to make individuals less inclined to participate in health surveys (59). On that note, we believe the students to be under the most pressure, and to experience the least beneficial health and wellbeing, during the data collection phase to be underrepresented in the sample. On the other hand, the gender distribution in the sample appears to mirror the population, thereby strengthening the notion that gender differences could be replicated. Finally, the reliance on self-report measures on constructs where cultural and social desirability are likely to be at play may on the one hand cast light on the cultural influence on impostor feelings, on the other make it harder to interpret results. However, the gender representation of the study participants is proportional to the gender distribution of the invited students. Due to the cross-sectional nature, longitudinal analysis of students’ impostor feelings and psychological distress is unattainable. The survey did not account for variables such as students’ examination status or transitional phases during the program. This could have provided valuable insights, given Clance et al.’s suggestion that IP is triggered by stressful events. Furthermore, the study did not address how IP relates to personality and perfectionism (14), aspects that could have contributed to valuable information.

## Conclusions and implications

This study demonstrates that IP could be a significant burden to Swedish university students in both the medical and social science fields. The findings indicate a relationship between IP and perceived stress and anxiety. Women report higher levels of IP, perceived stress, and anxiety compared to the males in the study. The results underscore the importance of further exploring IP and its link to psychological distress.

Subsequent studies should employ a longitudinal framework that comprehensively tracks the development of IP over an entire academic program and the transition into professional life (60). An initial step would involve investigating the prevalence of IP and its correlation with psychological distress among trainee professionals to assess any possible shifts in the impact of IP during the transition to professional life. Furthermore, studies on interventions aiming at mitigating IP among university students in the Swedish context should be prioritized. Previously suggested strategies to mitigate IP among students include coaching (61) and online information and training (62). In addition, qualitative methodologies are essential to deepen the understanding of how impostor feelings are experienced (63), an approach that has not yet been applied in the Swedish context.

## Data Availability

The raw data supporting the conclusions of this article will be made available by the authors, without undue reservation.
